# Practice variation in opioid prescribing for non-cancer pain in Dutch primary care: A retrospective database study

**DOI:** 10.1371/journal.pone.0282222

**Published:** 2023-02-24

**Authors:** G. A. Kalkman, C. Kramers, R. T. van Dongen, H. J. Schers, R. L. M. van Boekel, J. M. Bos, K. Hek, A. F. A. Schellekens, F. Atsma

**Affiliations:** 1 Department of Clinical Pharmacy, Canisius-Wilhelmina Hospital, Nijmegen, The Netherlands; 2 Department of Pharmacology and Toxicology, Radboud University Medical Center, Radboud Institute for Health Sciences, Nijmegen, The Netherlands; 3 Department of Anesthesiology, Radboud University Medical Center, Pain and Palliative Care, Nijmegen, The Netherlands; 4 Pain Department, Canisius-Wilhelmina Hospital, Nijmegen, The Netherlands; 5 Department of Primary and Community Care, Radboud University Medical Center, Nijmegen, The Netherlands; 6 Nivel, Netherlands Institute for Health Services Research, Utrecht, The Netherlands; 7 Department of Psychiatry, Radboud University Medical Center, Nijmegen, The Netherlands; 8 Radboud University Medical Center, Radboud Institute for Health Sciences, Scientific Center for Quality of Healthcare (IQ Healthcare), Nijmegen, The Netherlands; University of Technology Sydney, AUSTRALIA

## Abstract

**Background:**

Prescription opioid use has increased steadily in many Western countries over the past two decades, most notably in the US, Canada, and most European countries, including the Netherlands. Especially the increasing use of prescription opioids for chronic non-cancer pain has raised concerns. Most opioids in the Netherlands are prescribed in general practices. However, little is known about variation in opioid prescribing between general practices. To better understand this, we investigated practice variation in opioid prescribing for non-cancer pain between Dutch general practices.

**Methods:**

Data from 2017–2019 of approximately 10% of all Dutch general practices was used. Each year included approximately 1000000 patients distributed over approximately 380 practices. The primary outcome was the proportion of patients with chronic (>90 days) high-dose (≥90 oral morphine equivalents) opioid prescriptions. The secondary outcome was the proportion of patients with chronic (<90 oral morphine equivalents) opioid prescriptions. Practice variation was expressed as the ratio of the 95th/5th percentiles and the ratio of mean top 10/bottom 10. Funnel plots were used to identify outliers. Potential factors associated with unwarranted variation were investigated by comparing outliers on practice size, patient neighbourhood socioeconomic status, and urbanicity.

**Results:**

Results were similar across all years. The magnitude of variation for chronic high-dose opioid prescriptions in 2019 was 7.51-fold (95%/5% ratio), and 15.1-fold (top 10/bottom 10 ratio). The percentage of outliers in the funnel plots varied between 13.8% and 21.7%. Practices with high *chronic* *high-dose* *opioid prescription proportions* were larger, and had more patients from lower income and densely populated areas.

**Conclusions:**

There might be unwarranted practice variation in chronic high-dose opioid prescriptions in primary care, pointing at possible inappropriate use of opioids. This appears to be related to socioeconomic status, urbanicity, and practice size. Further investigation of the factors driving practice variation can provide target points for quality improvement and reduce inappropriate care and unwarranted variation.

## Introduction

Prescription opioid use has increased steadily in the past two decades, most notably in the United States (US), Canada, and many European countries [1] including the Netherlands [2, 3]. In the US, increased opioid prescribing contributed to a sharp increase in opioid-related harm (the so-called “opioid epidemic”) [4]. Though in the Netherlands levels of opioid-related harm are not comparable to the US, concerns have been raised about the increased medical use of prescription opioids, mainly oxycodone [2, 3] In The Netherlands, the percentage of inhabitants being prescribed an opioid nearly doubled from 4.1% in 2008 to 7.5% in 2017 [3], after which it stabalised [5]. This can be attributed to increased prescribing by both general practitioners and medical specialists, however general practitioners prescribe the vast majority of opioids [6]. Most of these opioids are being prescribed for non-cancer pain [7] and evidence for their effectiveness in this type of pain is lacking [8]. In addition, chronic opioid use increases the risk for opioid addiction, overdose, and mortality, especially when high doses are prescribed [9].

Previous research has shown that large variation in opioid prescribing exists between geographical regions in the US [10], general practices in England [11], hospitals in the US [12], and physicians within the same hospital in the US [13]. This variation may be an important signal for the inappropriate use of opioids. When looking at medical practice variation, a distinction should be made between warranted and unwarranted sources [14]. In general, variation attributed to factors related to disease incidence or patient preferences is considered warranted because it affects the need for treatment. In contrast, variation attributed to provider-related factors or unclear clinical standards is considered unwarranted [14]. Large unwarranted variation may indicate over- or underuse of healthcare services and may provide an important signal for suboptimal care [15]. In-depth insight in these patterns within European countries is needed and will provide input for targeted strategies to improve quality of care.

Until now, most research on variation in opioid prescribing has been conducted in the United States and is unlikely to be generalizable to European countries due to differences in health care organisation and opioid situation. For example, a general practitioner is the cornerstone of healthcare in many European countries and functions as a gatekeeper to specialist care [16]. More than 80% of opioids in the Netherlands are prescribed by general practitioners [6]. In contrast, opioid prescribing in the US is more fragmented. Primary care physicians are also the top opioid prescribers in the US, but they only account for a third of all opioids [17]. To our knowledge, the only in-depth studies examining opioid prescribing in primary care were conducted in the United Kingdom [11, 18]. However, investigating practice variation in opioid prescribing was not the main objective of these studies.

In the present study we therefore investigated practice variation in chronic opioid prescribing for non-cancer pain by general practitioners in the Netherlands. The primary outcome measure was the proportion of patients with chronic high-dose (≥90 oral morphine equivalents [OME]) opioid prescriptions, and the secondary outcome was the proportion of patients with chronic <90 OME opioid prescriptions. Opioid use for a period of 90 days or longer was considered chronic [19]. These outcomes were chosen because they represent different types of opioid prescribing according to the Dutch College of General Practitioners (NHG) guideline for Pain treatment [20]. It states that short term treatment with opioids for non-cancer pain can be initiated when non-opioid treatments provide insufficient relief. Chronic treatment with opioids should only be initiated when patients clearly benefit from opioids and the benefits outweigh the harms. Daily doses above 90 OME should be avoided to limit potential harm. To our knowledge, practice variation in opioid prescribing in this detail, by using outcomes that reflect duration, dose, and indication, has not yet been examined. Furthermore, a novelty of this study is that we aimed to understand the variation by comparing characteristics of high and low prescribing practices.

## Methods

### Data source

We used pseudonymised electronic health record data from Dutch general practices collected in the Nivel Primary Care Database (Nivel-PCD) for the calendar years 2017 to 2019. Nivel-PCD collects longitudinal data on patient characteristics, drug prescriptions, and disease episodes from approximately 10% of all general practices in the Netherlands [21]. Data belonging to the same patient within a general practice can be uniquely identified. Participating general practices were geographically spread across the Netherlands and their patients were representative for the entire Dutch population [22]. Prescriptions were coded according to the Anatomical Therapeutic Chemical [23] (ATC) classification system and information on the specific drug formulation, start date and dose is available. Disease episodes were coded according to the International Classification of Primary Care version 1 (ICPC-1) [24].

The use of personal data for research purposes in the Netherlands is regulated under the Dutch Medical Treatment Contracts Act (WGBO). The WGBO stipulates that explicit consent is not required if a) requesting consent is not reasonably possible (if for example the patient is deceased) or- if b) the request for permission cannot reasonably be expected from the caregiver. The latter can refer to situations in which too great effort an effort is needed from health care providers, or when asking for permission would lead to a selective response. However, data collection should take place taking into account all possible organizational and technical measures needed. In addition, the Medical Research Involving Human Subjects Act (WMO), stipulates that approval by one of the national medical ethical committees is required only if the research involves humans subjected to actions or if rules of behavior are imposed on them. This is not the case in our study. General practices that participate in Nivel-PCD are contractually obliged to: (i) inform their patients about their participation in Nivel-PCD and (ii) to inform patients about the option to opt-out for inclusion of their data in the database. Data were pseudonymized before leaving the health care organization’s premises and did not comprise any directly identifying personal information such as names, addresses and citizen service number [23]. Neither obtaining informed consent from patients nor approval by a medical ethics committee is obligatory for observational studies containing no directly identifiable data (Dutch Civil Law, Article 7: 458). The study was approved according to the governance code of Nivel-PCD under number: NZR-00319.034, and all legally required technical and organizational measures were applied to avoid real life identification of subjects.

### Population

For each year, all general practices were included for which high-quality data was available. The quality criteria were: 1) data covering a period of at least 46 weeks, 2) at least 85% of prescriptions had a valid ATC code, 3) at least 75% of contacts had a valid ICPC code, 4) at least 500 patients were registered, and 5) at least 80% of a practice’s patients were registered with that practice for the entire year. These criteria exclude general practices with poor or incomplete data registration.

From these practices, all patients were included who 1) were at least 20 years old, 2) were registered with their practice for the entire year, and 3) did not have a cancer diagnosis (e.g., not having a cancer diagnosis that was active in the year of analysis). Flowcharts describing the number of patients included and excluded are presented in S1 Fig.

### Definitions and variables

Opioid prescriptions were identified using ATC codes N02A (Opioids) and N07BC (Drugs used in opioid dependence). Codeine was excluded, since it is predominantly used for cough. OME’s were calculated based on the type of opioid, the amount prescribed, and the route of administration. The OME conversion ratio’s from Nielsen et al. [25] were used when possible.

The primary outcome was the proportion of patients of the total general practice population receiving a long-term high-dose opioid prescription, which was defined as opioid prescriptions covering a period of at least 90 consecutive days with an average daily dose of 90 mg OME or greater. The secondary outcome was the proportion of patients within general practices with chronic opioid use in general (without focussing on high douse use), which defined as opioid prescriptions covering a period of at least 90 consecutive days with an average daily dose of <90 OME. Patients with opioid prescription patterns that met both the definition for chronic and chronic high-dose in a single year were only classified as chronic high-dose. Details on the method for identifying patients with chronic (high-dose) opioid prescriptions can be found in S1 Text.

Age was calculated at the first of January of a year and was provided in 5-year age groups. The number of chronic diseases was defined as the number of registered patient diagnoses that were present in a predefined list of chronic diseases [26].

### Statistical analysis

All analyses were performed separately for each year to assess robustness of the analyses over separate years.

### Descriptive statistics

Basic descriptive statistics for patients and practices are shown separately for all years: total number of patients and practices, number of patients per practice (mean, minimum, and maximum), number of observed outcomes, age and sex distribution, and mean number of chronic diseases per patient.

### Practice variation analyses

Proportions of patients with chronic use and chronic high-dose use within general practices were indirectly standardized for patient age, sex, and number of chronic diseases. To do this, observed (unadjusted) proportions were calculated for each practice separately. Subsequently, a logistic regression model was performed to predict each patient’s probability of experiencing an outcome (chronic use and chronic high-dose) based on the adjustment factors and to calculate the expected proportions of chronic use and chronic high-dose use per practice. The logistic regression was performed separately for both outcomes (chronic use and chronic high-dose use). The indirectly standardized proportion was obtained by calculating the observed/expected ratio per practice and multiplying this ratio by the overall proportion. To avoid standardized proportions of zero, only practices with at least one patient with the outcome were included. Practices without any patients with the outcome were therefore reported separately.

### Magnitude of variation

For each outcome (adjusted and unadjusted), a 95%/5% percentile ratio was calculated per year to quantify the amount of variation between practices. Similarly, a mean top 10/bottom 10 ratio was calculated.

Funnel plots were constructed to graphically represent practice variation on the outcome measures. The observed-expected ratios per practice were plotted against the expected number of patients with the outcome, and control limits (95% and 99.8%) were drawn around the target value (O/E = 1) [27]. Practices outside of the control limits are considered outliers. To account for uncontrolled variation control limits were adjusted for overdispersion by the method of Spiegelharter, which is a default functionality of the FunnelplotR package in R. [27, 28].

### Understanding variation

To interpret the variation, general practices that were outliers in the funnel plot (both above and below control limits) or had no outcome (i.e. had extreme values) for chronic high-dose opioid prescriptions in 2019 were compared with respect to practice size, patient socioeconomic status (SES), and urbanicity. These factors were chosen because they are known to be related to opioid prescribing [11] and were present in our dataset. Urbanicity (in house addresses per km2) and SES (in percentage of people with a high or low income within a zip code area) were based on the first 4 digits of a patient’s zip code and were retrieved from Statistics Netherlands (CBS). Data from 2017 was used, as this was the most recent dataset containing SES information. Per group (no outcome, low, and high outliers), mean practice size, mean adjusted proportions of chronic use, mean high-/low-income percentages, and mean urbanicity were reported.

All statistical analyses were done using R version 4.0.1. [29]. The package FunnelPlotR was used to construct the funnelplots, which includes a command to adjust for overdispersion by the method of Spiegelharter [30].

## Results

Descriptive practice and patient characteristics per year are shown in Table 1. Overall, differences between descriptives across years were clinically unimportant. Flowcharts describing the number of patients included and excluded are presented in S1 Fig. An OME value could be calculated for 98.3% of all opioid prescriptions. In 2019 the overall percentages of patients with chronic and chronic high-dose prescriptions were 1.43% and 0.16% respectively.

**Table 1:** Basic patient and practice characteristics per year.

	2017	2018	2019
Patients (n)	1 052 288	1 097 670	1 024 466
Practices (n)	378	388	361
Patients included per practice			
Mean	2 784	2 829	2 838
Min—max	855–12 143	943–12 177	1 093–12 262
Opioid prescriptions			
Chronic <90 OME	17 199 (1.63%)	17 731 (1.62%)	14 614 (1.43%)
Chronic high-dose (≥90 OME)	1 816 (0.17%)	1 830 (0.17%)	1 599 (0.16%)
Male (%)	516 812 (49.1%)	539 766 (49.2%)	504 890 (49.3%)
Age			
20–39	326 132 (31.0%)	339 340 (30.9%)	313 217 (30.6%)
40–59	397 393 (37.8%)	410 033 (37.4%)	376 860 (36.8%)
60–79	274 619 (26.1%)	291 456 (26.6%)	278 962 (27.2%)
80+	54 144 (5.2%)	56 841 (5.2%)	55 427 (5.4%)
Number of chronic diseases			
0	495 674 (47.1%)	589 126 (53.7%)	417 577 (40.8%)
1	231 347 (22.0%)	208 323 (19.0%)	248 217 (24.2%)
2	132 661 (12.6%)	121 042 (11.0%)	144 812 (14.1%)
3	78 908 (7.50%)	72 522 (6.6%)	86 285 (8.4%)
≥4	113 698 (10.8%)	106 657 (9.7%)	127 575 (12.5%)

OME = Oral morphine equivalents

Table 2 shows the variation (95/5% and mean top 10/bottom 10) for the primary and secondary outcomes. The number of practices without any patients with chronic high-dose (≥90 OME) opioid prescriptions were 27 (7%), 31 (8%), and 34 (9%) in 2017, 2018, and 2019 respectively. There were no practices without chronic <90 OME opioid prescriptions.

**Table 2:** Variation scores for chronic high-dose (≥90 OME) use and chronic <90 OME prescriptions across years. The proportions on which the ratios are based is shown in brackets.

	95%/5% ratio^b^				mean top 10/mean bottom 10 ratio^c^		
	2017	2018	2019		2017	2018	2019
Chronic <90 OME							
Unadjusted	3.9	4.1	3.9		9.4	9.9	9.4
	(= 2.78% / 0.72%)	(= 2.88% / 0.71%)	(= 2.42% / 0.63%)		(= 3.64% / 0.39%)	(= 3.70% / 0.37%)	(= 3.41% / 0.36%)
Adjusted^a^	4.4	4.4	4.2		11.5	9.8	9.4
	(= 3.06% / 0.69%)	(= 3.22% / 0.74%)	(= 2.61% / 0.62%)		(= 4.90% / 0.43%)	(= 4.74% / 0.49%)	(= 3.75% / 0.40%)
Chronic high-dose (≥90 OME)							
Unadjusted	9.0	8.4	6.9		17.1	19.2	14.8
	(= 0.42% / 0.05%)	(= 0.39% / 0.05%)	(= 0.34% / 0.05%)		(= 0.52% / 0.03%)	(= 0.53% / 0.03%)	(= 0.53% / 0.04%)
Adjusted^a^	9.9	9.9	7.5		22.6	20.9	15.1
	(= 0.44% / 0.05%)	(= 0.44% / 0.04%)	(= 0.35% / 0.05%)		(= 0.67% / 0.03%)	(= 0.57% / 0.03%)	(= 0.52% / 0.04%)

The proportions on which the ratios are based is shown in brackets.

^a^ Adjusted for ages, sex, cancer, and number of chronic diseases

^b^ 95%/5% ratio was calculated by dividing the proportion of the 95^th^ percentile general practice by the proportion of the 5^th^ percentile general practice

^c^ mean top 10/mean bottom 10 ratio was calculated by dividing the mean proportion of the top 10 general practices by the mean proportion of bottom 10 general practices

In 2019, the adjusted variation for chronic high-dose (≥90 OME) prescriptions was 7.51, meaning there was a 7.51-fold variation in proportions between the 95^th^ percentile general practices and the 5^th^ percentile general practices. This variation was slightly larger in previous years, with a 9.90-fold and 9.93-fold variation in 2017 and 2018 respectively. In 2019, there was a 15.1-fold variation between the highest 10 general practices and the lowest 10 general practices in the proportion of patients with chronic high-dose prescriptions. In 2017 and 2018, there were a 22.6-fold and 20.9-fold variation, respectively.

**Fig 1:**
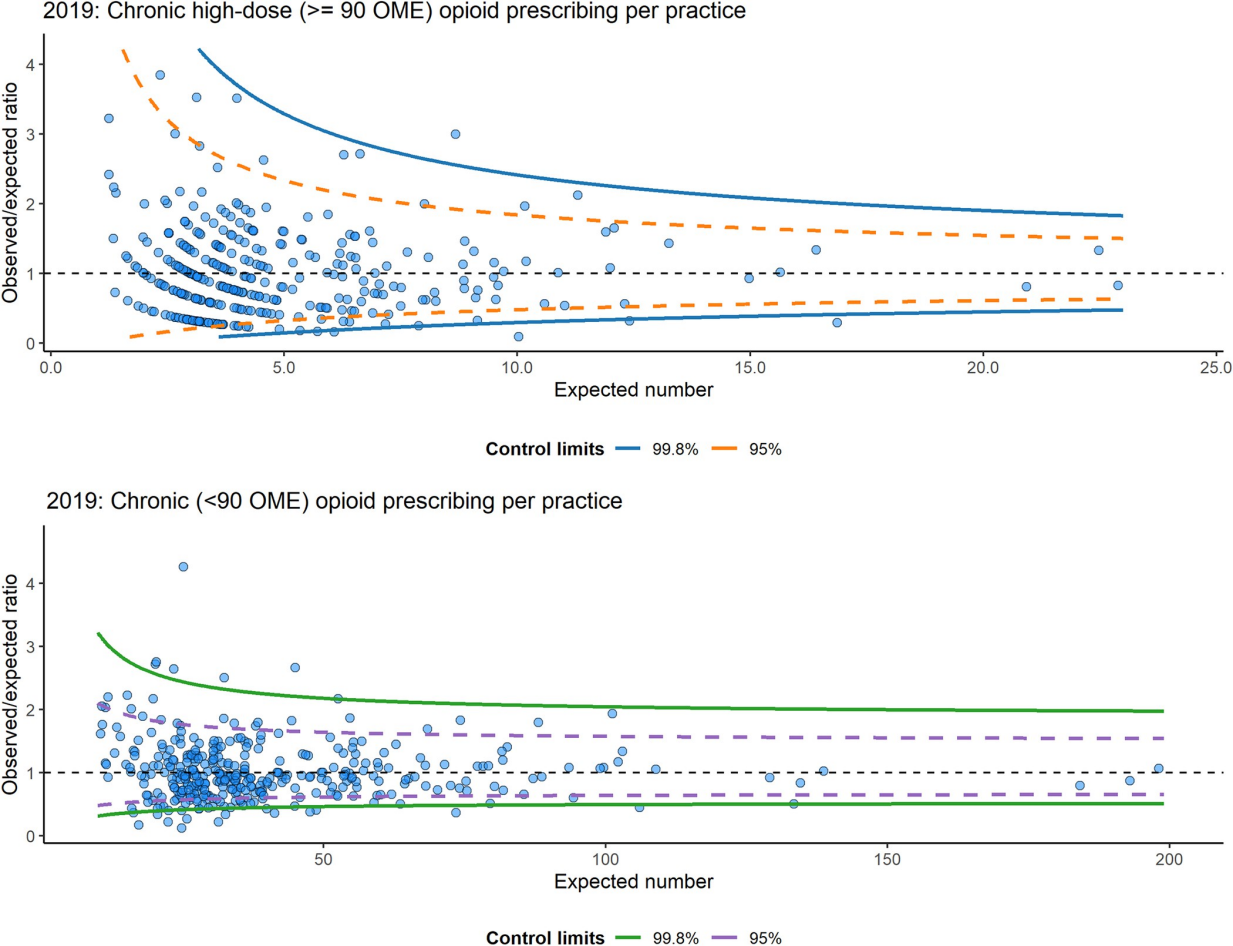
Funnel plots for both outcomes for 2019. Control limits for the outcome chronic < 90 OME were corrected for overdispersion. The horizontal black dotted line (O/E = 1) represents the target value where the observed proportion equals the expected proportion based on case-mix. Each dot represents a single practice. Variation between practices that lie between the 95% control limits is considered to be caused by random variation. Practices above or below the 95% control limits are considered outliers and were categorised as either high or low prescribers, respectively.

Funnel plots for both outcomes (chronic high-dose (≥90 OME) and chronic <90 OME opioid prescriptions) are shown in Fig 1 for the year 2019. Figs for 2018 and 2017 were comparable and can be found in S2 Fig. Overdispersion was present and corrected for in all years and outcomes, except for chronic high-dose (≥90 OME) prescriptions in 2019. The percentage of outliers was similar across all years and outcomes, varying between 13.8% and 21.7% (Table 3), with most outlying practices being low prescribers.

**Table 3:** Number of outliers in the funnel plots for all years and outcomes.

	2017	2018	2019
Outliers **outside** 95% overdispersed limits			
Chronic <90 OME	74 (19.6%)	77 (19.8%)	71 (19.7%)
Chronic high-dose (> = 90 OME)	63 (17.9%)	61 (17.1%)	45 (13.8%)
Outliers **above** 95% overdispersed limits			
Chronic <90 OME	22 (5.8%)	28 (7.2%)	24 (6.7%)
Chronic high-dose (> = 90 OME)	13 (3.7%)	6 (1.7%)	14 (4.3%)
Outliers **below** 95% overdispersed limits			
Chronic <90 OME	52 (13.8%)	49 (12.6%)	47 (13.0%)
Chronic high-dose (> = 90 OME)	50 (14.2%)	55 (15.4%)	31 (9.5%)

Table 4 shows a comparison of outlying practices with respect to the proportion of patients with chronic high-dose opioid prescriptions. Overall, practices with a high proportion of patients with *chronic* *high-dose* *opioid prescriptions* were the larger general practices, had more patients with a lower SES, and from higher urbanicity areas. In addition, these practices also prescribed more chronic <90 OME opioid therapy, compared to practices with a low proportion of chronic high-dose prescriptions.

**Table 4:** Comparison of outlying practices in 2019.

	No patients with chronic high-dose (≥90 OME) prescriptions	Low outliers (below 95% control limits)	High outliers (above 95% control limits)
Practice			
N	34	31	14
Median practice size (IQR)	3 016	4 684	5 760
	(2 348–3 108)	(2 790–6 290)	(2 696–7 675)
Median proportion of chronic <90 OME prescriptions (IQR)	1.05%	0.95%	2.22%
	(0.68%– 1.29%)	(0.70%– 1.08%)	(1.91%– 2.60%)
Median proportion of chronic > = 90 OME prescriptions (IQR)	-	0.046%	0.46%
		(0.042% - 0.051%)	(0.37% - 0.51%)
Patient			
Median percentage low income (IQR)	34%	32%	44%
	(29% - 38%)	(26% - 36%)	(35% - 55%)
Median percentage high income (IQR)	24%	26%	15%
	(19% - 28%)	(20% - 34%)	(9% - 20%)
Median urbanicity in addresses / km2 (IQR)	1 242	1 334	1 688
	(361–1 461)	(720–1 621)	(981–1 971)

Practices with a high proportion of patients with high-dose opioid prescriptions versus practices with a low proportion of patients with high-dose opioid prescriptions. Practices were grouped into three categories: practices without any chronic high-dose prescriptions, and low and high outliers on the funnel plot.

## Discussion

In this study we explored practice variation in the proportion of patients within a general practices with opioid prescriptions for chronic non-cancer pain in Dutch primary care. In 2019 there was a 7.51-fold (95%/5%) practice variation in adjusted proportions of patients with chronic high-dose (≥90 OME) opioid prescriptions. For chronic <90 OME prescriptions, the adjusted variation was 4.19-fold. This finding is further supported by the larger than expected number of outliers (13.8% of practices in 2019). For 2017 and 2018 results were comparable, showing that the variation is consistent across the years. Moreover, the variation could not be attributed to sources of warranted variation (age, sex, and number of chronic diseases).

According to a recent systematic review by Sutherland et al., unwarranted variation can be explained using three domains: agency, evidence, and capacity [31]. Variation explained by medically irrelevant patient characteristics is seen as unwarranted from an agency perspective. For example, SES might influence the choice of pain therapy. Many non-pharmacological pain therapies (e.g. physical therapy) are not always covered by the Dutch mandatory basic health insurance and often require additional insurance. Opioids are always covered and might therefore be preferred by patients with a lower SES. Our data suggests this might be the case, as practices with a high proportion of chronic high-dose opioid prescriptions had more patients from low-income areas. Our results are in line with research by Curtis *et al*., who found wide variation in opioid prescribing in England, which was related to practice size, rurality, and poverty [11]. Research has also shown that lower SES is associated with increased prevalence of pain [32] and ineffective coping styles [33] which could drive demand for opioids in this population. Although this might partly explain the variation, it should be questioned if this justifies increased opioid prescribing in this population.

Unwarranted variation may also emerge when clinical practice is not supported by the evidence. For example, differences in the interpretation of the NHG guideline for Pain management could result in important differences between practices. The guideline states that acute non-cancer pain can be treated with opioids when other treatments provide insufficient relief and daily functioning is severely inhibited. Chronic pain management with opioids for non-cancer pain should only be initiated when patients clearly benefit from opioids and the benefits outweigh the (potential) harms [20]. However, the guideline is unclear on what insufficient relief means, how to (objectively) measure this, and what to consider when weighing the harms and benefits. Different general practitioners might value the effectiveness of opioid therapy differently and have different views on their benefits and risks. Research by Desveaux et al. showed that opioid prescribing by family physicians in Ontario, Canada was highly influenced by their personal beliefs on opioids and on their own capability to safely prescribe them. The subjective nature of opioid prescribing is further supported by Martens *et al*. [34] who found that Dutch physicians in long-term geriatric care based their choices for opioid therapy almost exclusively on personal experience, rather than guidelines or scientific evidence.

An important goal of practice variation research is to define target points to improve quality of care by decreasing unwarranted variation and by increasing appropriateness of care. First signalling and then explaining practice variation are the first steps towards achieving this goal [14]. However, explaining variation in healthcare utilisation and separating warranted from unwarranted variation remains challenging. Especially for chronic opioid prescribing, which requires balancing the benefits of therapy with the potential risks in collaboration with the patient [20]. In our study we corrected for several factors associated with warranted variation, and we compared outlying practices in an attempt to further grasp the variation we found. Even after correction the amount of variation remained similar, which suggests a high amount unwarranted variation. However, our data do not capture the many nuanced considerations that a physician might have when prescribing opioids. Future research is needed to further explain and understand the variation in opioid prescribing we found. Research using qualitative methods (e.g. in depth interviews and focus groups) that focusses on high and low opioid prescribers and their patients could reveal important additional information on how interpret the observed practice variation and to improve quality of care in patients with chronic pain. Different interpretation of guidelines, local policies, knowledge on pain therapy, beliefs on opioid therapy from both physicians and patients, the influence of SES, and views on non-opioid and non-pharmacological therapies should be investigated in more depth. In addition, future research should consider the quality of pain management in relation to opioid prescribing. A better understanding of the quality of pain management in high, average, and low prescribing practices would give insight into how much reduction in opioid prescribing is achievable without compromising quality of pain management. Especially the practices that prescribe no chronic high-dose opioids are interesting in this regard. However, optimising pain management should be the goal, not merely reducing the number of opioid prescriptions.

This study has strengths and limitations. Major strengths of this study are the use of a large, detailed dataset and the use of relevant outcome measures. The high level of detail in our dataset allowed us to use endpoints that distinguish between higher (≥90 OME) and lower (<90 OME) daily opioid doses. This substantially adds to the clinical relevance of our findings because higher daily doses are associated with higher risks (e.g. addiction and overdose) and are treated differently in (inter)national guidelines [9, 20]. This study also has several limitations. First, our data only contains prescriptions by general practitioners. Prescriptions originating from other sources (e.g., hospitals) are not included in our dataset. However, research has shown that most of the opioids in the Netherlands are prescribed by general practitioners, so the underestimation of opioid use is likely to be small [6]. Second, we measured chronic opioid prescribing within calendar years. This means that chronic opioid use that starts during the last 3 months of a year could not be identified. Since this approach was used within all practices and years, biased results about variation are unlikely. However, outcome proportions may be somewhat underestimated. Third, our data only uniquely identifies patients within a practice. Switching of patients between practices cannot be detected. Patients visiting multiple doctors at the same time (doctor shopping) is highly unlikely to occur, because in the Netherlands a patient can only be registered with one general practice which provides routine care and acts a gatekeeper to secondary care. It is therefore unlikely that our results were biased by this. Fourth, the number of included patients dropped slightly in 2019, compared to previous years. This can be explained by a smaller number of practices that participated in the Nivel PCD. Data quality of the participating practices was, to our knowledge, not negatively affected, as our inclusion criteria also include data quality criteria. Fifth, we excluded cancer patients based on a cancer diagnosis. However, coded registration of cancer diagnosis in Dutch general practices is known to be poor [35], possibly resulting in the inclusion of some patients with cancer in our study. This could potentially reduce the amount of variation in our study because we expect there is less variation in opioid prescribing for cancer related pain than for non-cancer pain. Finally, our analysis includes opioids from ATC group N07BC (drugs used in opioid dependence) as these drugs are often used for pain, especially in general practice. We have conducted a sensitivity analysis whereby we excluded this ATC group. The results are presented in S2 Text. The main analysis (magnitude of variation and funnel plots) remains identical. Our exploratory analysis comparing the outliers shows a difference in the results for urbanicity. The sensitivity analysis showed higher urbanicity for practices with low or no chronic high-dose opioid prescribing, compared to the original analysis. Research using qualitative methods might provide more insight in the role of urbanicity in opioid prescribing.

In conclusion, this is the first Dutch study investigating practice variation in several key measures of opioid prescribing, revealing large unwarranted variation. This may point at suboptimal care and inappropriate opioid prescribing practices. Further research should focus more in depth on the differences between high and low prescribing practices to further explain this variation, with special consideration given to the comparison of quality of pain management in low opioid prescribing practices versus high prescribing practices. Themes to investigate could be general practitioner’s beliefs on opioid prescribing and pain management, interpretation of clinical standards, the influence of physician workload, and the role of patient preferences. Ultimately, increasing knowledge about causes of unwarranted variation may provide target points for improvement of quality of care and tackling inappropriate opioid use in patients with chronic pain.

## Supporting information

S1 Text Identification of chronic (high-dose) opioid use. (DOCX)

S2 Text Sensitivity analysis where opioids with ATC N07BC were excluded. (DOCX)

S1 Fig Flowcharts describing the number of patients included and excluded per year. (DOCX)

S2 Fig Funnel plots for outcomes from 2017 and 2018. (DOCX)
