# Supplementary material for: Practice variation in opioid prescribing for non-cancer pain in Dutch primary care: A retrospective database study
Source: PLoS One. 2023 Feb 24;18(2):e0282222. doi: 10.1371/journal.pone.0282222 (PMC9955956; doi:10.1371/journal.pone.0282222)
Supplement: S1 Text — (DOCX) [file pone.0282222.s001.docx]

**S1. Text. Identification of chronic (high-dose) opioid use.**

Opioid prescriptions were identified using ATC codes N02A (Opioids) and N07BC (Drugs used in opioid dependence). Prescriptions for the same drug were considered repeat prescriptions when their active compound, formulation (eg. tablet, patch, capsule etc.) and strength were the same, and the prescription start dates were less than 90 days apart. The end date of a (repeat) prescription was defined as the start date of the next repeat prescription. The duration of the last prescription in a series of repeat prescriptions was calculated as the average duration of the previous prescriptions in the series. For non-repeat prescriptions, the duration was fixed at 14 days, which is the standard duration of a first prescription in the Netherlands. A similar method was previously used on the same database by Weesie et al.[26] For each prescription an average daily dose was calculated by dividing the amount prescribed (e.g. number of pills) by the prescription duration and converting this to OME. For each day, a patient’s total opioid use was calculated by summing the daily dose of all active prescriptions. Chronic high-dose use was defined as a period of 90 subsequent days on which the average daily dose exceeded 90 OME, and this dose was exceeded on a majority (>45) of days.
