# Supplementary material for: Practice variation in opioid prescribing for non-cancer pain in Dutch primary care: A retrospective database study
Source: PLoS One. 2023 Feb 24;18(2):e0282222. doi: 10.1371/journal.pone.0282222 (PMC9955956; doi:10.1371/journal.pone.0282222)
Supplement: S2 Text — (DOCX) [file pone.0282222.s002.docx]

**Sensitivity analysis where opioids with ATC N07BC were excluded.**

**Basic patient and practice characteristics per year:**

|  | **2017** | **2018** | **2019** |
| --- | --- | --- | --- |
| Patients (n) | 1 052 288 | 1 097 670 | 1 024 466 |
| Practices (n) | 378 | 388 | 361 |
| Outcome | | | |
| Chronic < 90 OME | 17 108 (1.63%) | 17 641 (1.61%) | 14 522 (1.42%) |
| Chronic high-dose (>= 90 OME) | 1 413 (0.13%) | 1 469 (0.13%) | 1 280 (0.12%) |

**Variation scores for chronic high-dose** **(≥90 OME) use and chronic <90 OME prescriptions across years.** The proportions on which the ratios are based is shown in brackets:

|  | **95%/5% ratio^b^** | | | | **mean top 10/mean bottom 10 ratio^c^** | | |
| --- | --- | --- | --- | --- | --- | --- | --- |
|  | **2017** | **2018** | **2019** | | **2017** | **2018** | **2019** |
| Chronic <90 OME | | | |  | | | |
| Unadjusted | 3.9 (=2.79% / 0.71%) | 4.1 (=2.87% / 0.71%) | 3.8 (=2.40% / 0.63%) | | 9.4 (=3.59% / 0.38%) | 10.0 (=3.66% / 0.37%) | 9.8 (=3.38% / 0.34%) |
| Adjusted^a^ | 4.4 (=3.07% / 0.69%) | 4.4 (=3.21% / 0.74%) | 4.2 (=2.61% / 0.62%) | | 11.4 (=4.82% / 0.42%) | 9.9 (=4.72% / 0.48%) | 9.6 (=3.73% / 0.39%) |
| Chronic high-dose (≥90 OME) | | | |  | | | |
| Unadjusted | 8.5 (=0.34% / 0.04%) | 7.3 (=0.32% / 0.04%) | 7.1 (=0.30% / 0.04%) | | 15.4 (=0.45% / 0.03%) | 17.7 (=0.45% / 0.03%) | 17.3 (=0.46% / 0.03%) |
| Adjusted^a^ | 9.5 (=0.37% / 0.04%) | 9.6 (=0.39% / 0.04%) | 7.8 (=0.31% / 0.04%) | | 18.5 (=0.55% / 0.03%) | 20.0 (=0.53% / 0.03%) | 16.8 (=0.45% / 0.03%) |

The proportions on which the ratios are based is shown in brackets.
^a^ Adjusted for ages, sex, cancer, and number of chronic diseases
^b^ 95%/5% ratio was calculated by dividing the proportion of the 95^th^ percentile general practice by the proportion of the 5^th^ percentile general practice
^c^ mean top 10/mean bottom 10 ratio was calculated by dividing the mean proportion of the top 10 general practices by the mean proportion of bottom 10 general practices

**Number of outliers in the funnel plots for all years and outcomes:**

|  | **2017** | **2018** | **2019** |
| --- | --- | --- | --- |
| Outliers outside 95% overdispersed limits | | | |
| Chronic < 90 OME | 75 (19.8%) | 79 (20.4%) | 72 (19.9%) |
| Chronic high-dose (>= 90 OME) | 51 (15.2%) | 54 (15.7%) | 40 (12.7%) |
| Outliers above 95% overdispersed limits | | | |
| Chronic < 90 OME | 23 (6.1%) | 28 (7.2%) | 25 (6.9%) |
| Chronic high-dose (>= 90 OME) | 24 (7.1%) | 19 (5.5%) | 16 (5.1%) |
| Outliers below 95% overdispersed limits | | | |
| Chronic < 90 OME | 52 (13.8%) | 51 (13.1%) | 47 (13.0%) |
| Chronic high-dose (>= 90 OME) | 27 (8.0%) | 35 (10.2%) | 24 (7.6%) |

**Comparison of outlying practices in 2019.**

|  | **No patients with chronic high-dose (≥90 OME) p**rescriptions | **Low outliers (below 95% control limits)** | **High outliers (above 95% control limits)** |
| --- | --- | --- | --- |
| Practice | | | |
| N | 46 | 24 | 16 |
| Median practice size (IQR) | 2 966 (2 360 - 3 126) | 5 592 (3 384 - 7 789) | 5 841 (3 016 - 7 916) |
| Median proportion of chronic < 90 OME prescriptions (IQR) | 1.12% (0.74% - 1.33%) | 0.94% (0.77% - 1.08%) | 2.22% (1.91% - 2.58%) |
| Median proportion of chronic >= 90 OME rate prescriptions (IQR) |  | 0.04% (0.03% - 0.04%) | 0.37% (0.30% - 0.43%) |
| Patient | | | |
| Median percentage low income (IQR) | 36% (29% - 44%) | 35% (26% - 43%) | 43% (32% - 54%) |
| Median percentage high income (IQR) | 22% (16% - 28%) | 25% (17% - 34%) | 15% (9% - 20%) |
| Median urbanicity in addresses / km2 (IQR) | 1 578 (370 - 1 775) | 2 105 (1 031 - 2 382) | 1 585 (981 - 1 796) |
