# Supplementary figures and images for: Practice variation in opioid prescribing for non-cancer pain in Dutch primary care: A retrospective database study
Source: PLoS One. 2023 Feb 24;18(2):e0282222. doi: 10.1371/journal.pone.0282222 (PMC9955956; doi:10.1371/journal.pone.0282222)

**S2. Fig. Flowcharts describing the number of patients included and excluded per year.**

2017
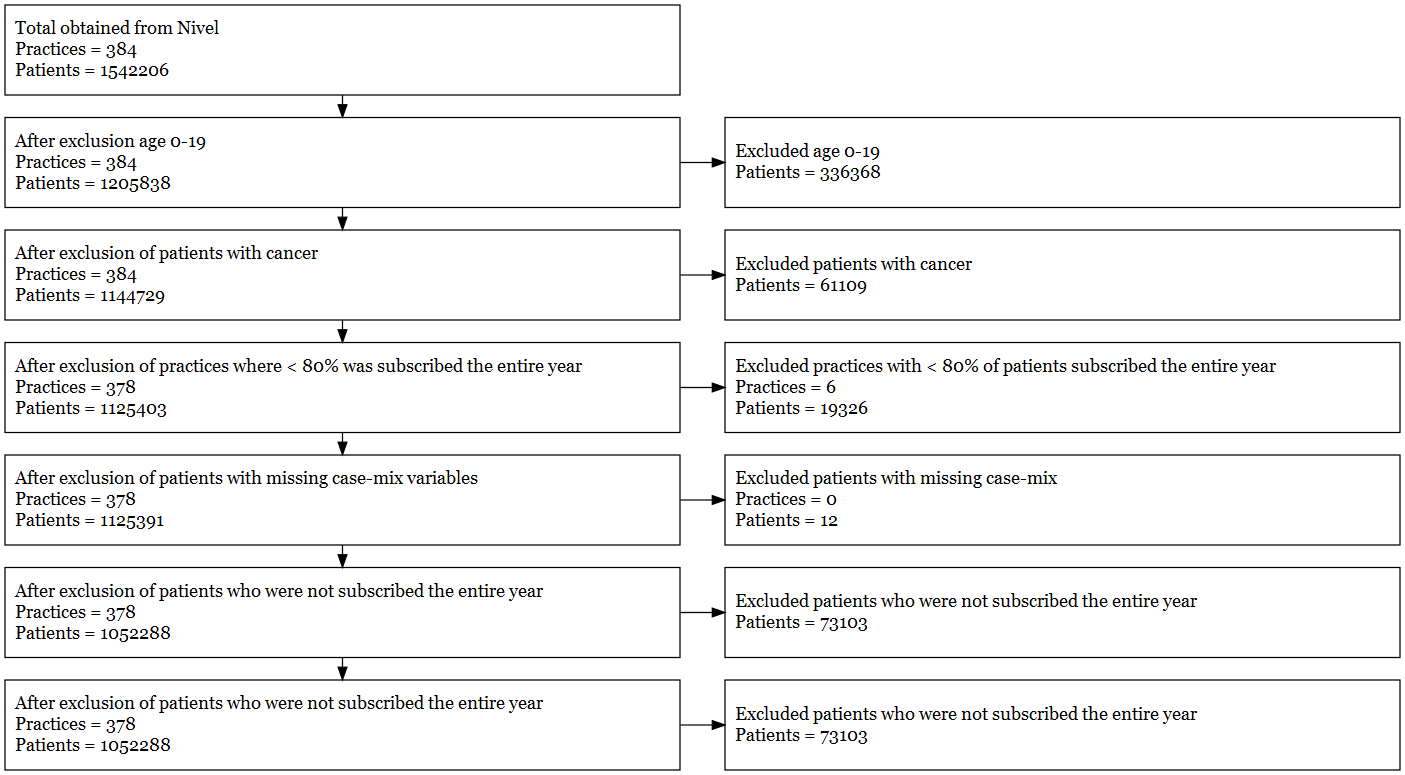


2018


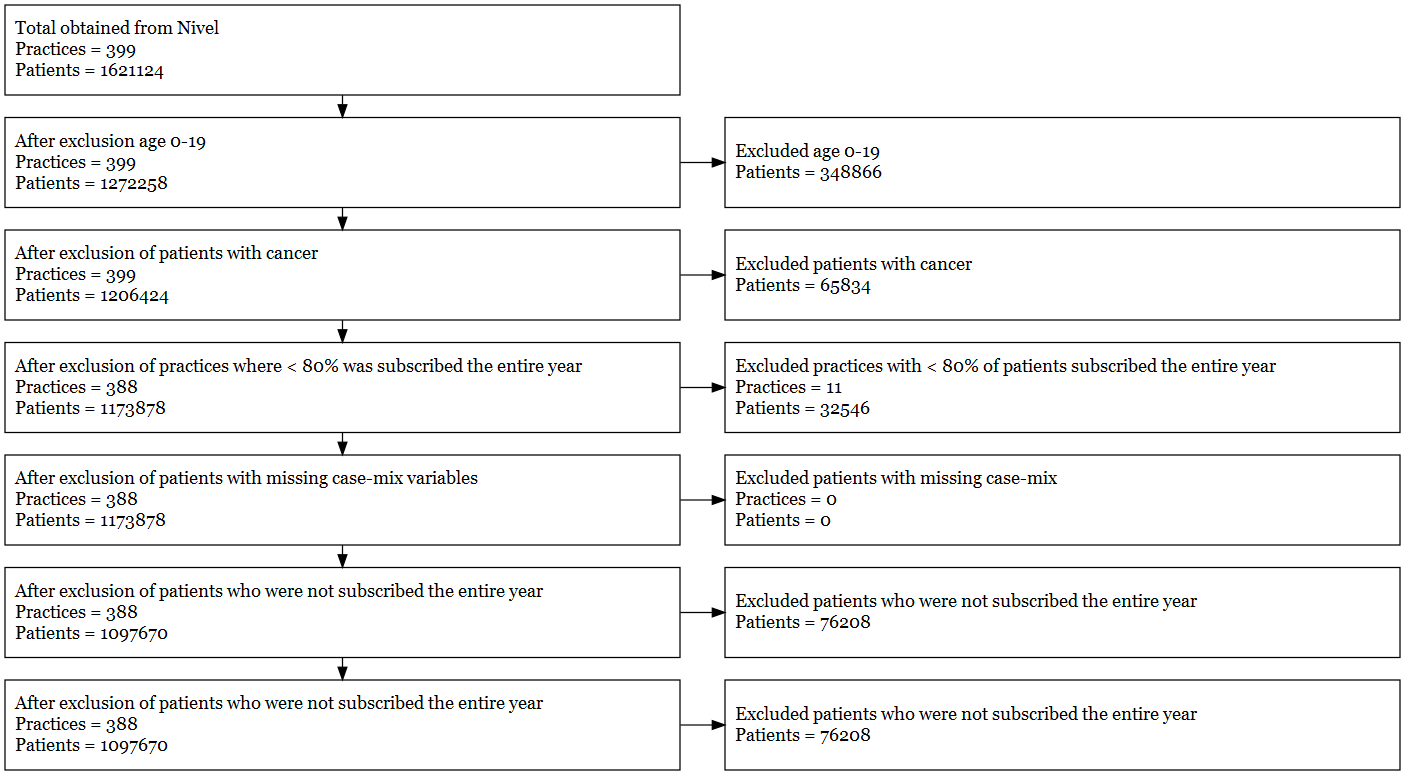


2019
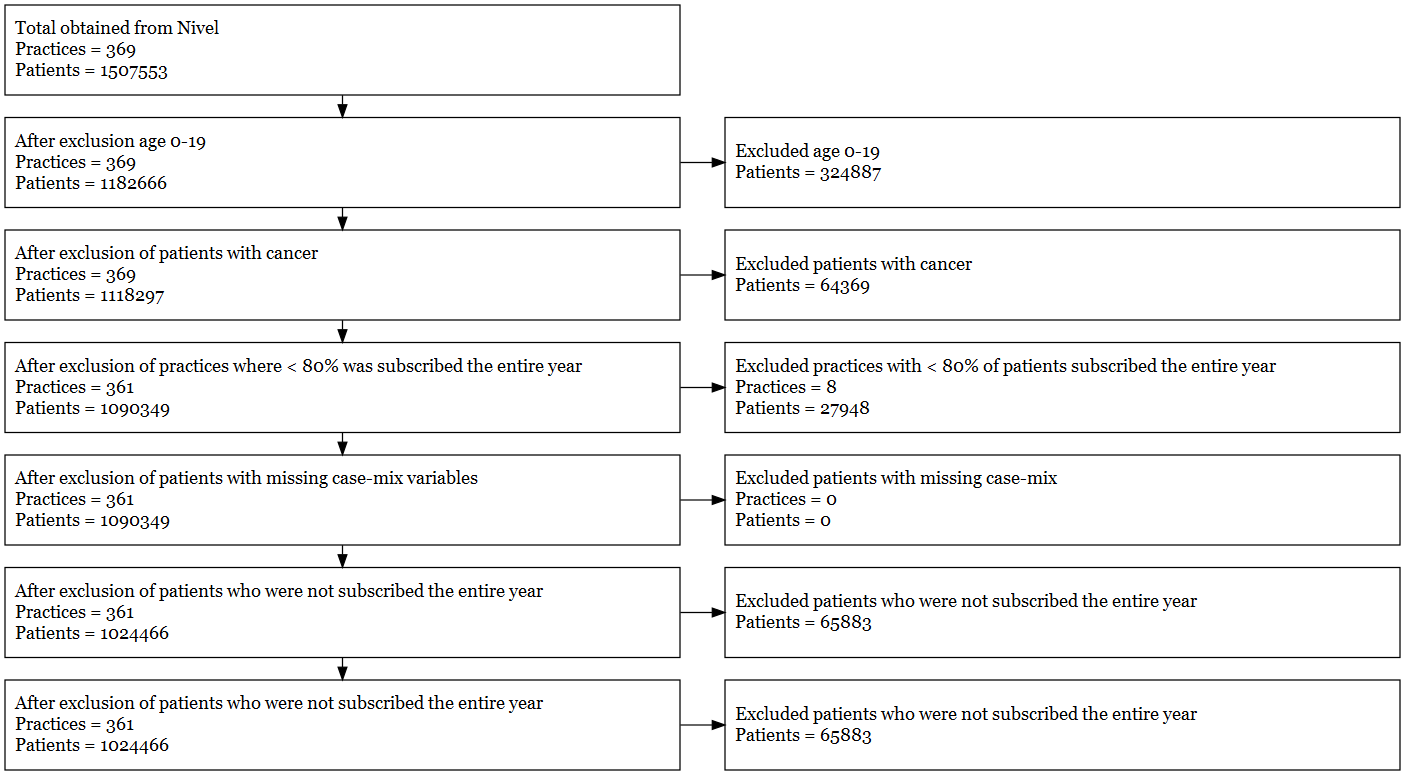

Supplement: S1 Fig — (DOCX) [file pone.0282222.s003.docx]
