# Supplementary material for: Practice variation in opioid prescribing for non-cancer pain in Dutch primary care: A retrospective database study
Source: PLoS One. 2023 Feb 24;18(2):e0282222. doi: 10.1371/journal.pone.0282222 (PMC9955956; doi:10.1371/journal.pone.0282222)

**S3. Fig. Funnel plots for outcomes from 2017 and 2018.**

Outcome rates were adjusted for age, sex, and number of chronic diseases. Control limits were corrected for overdispersion. The horizontal black dotted line (O/E = 1) represents the target value where the observed outcome rate equals the adjusted rate based on case-mix. Each dot represents a single practice.


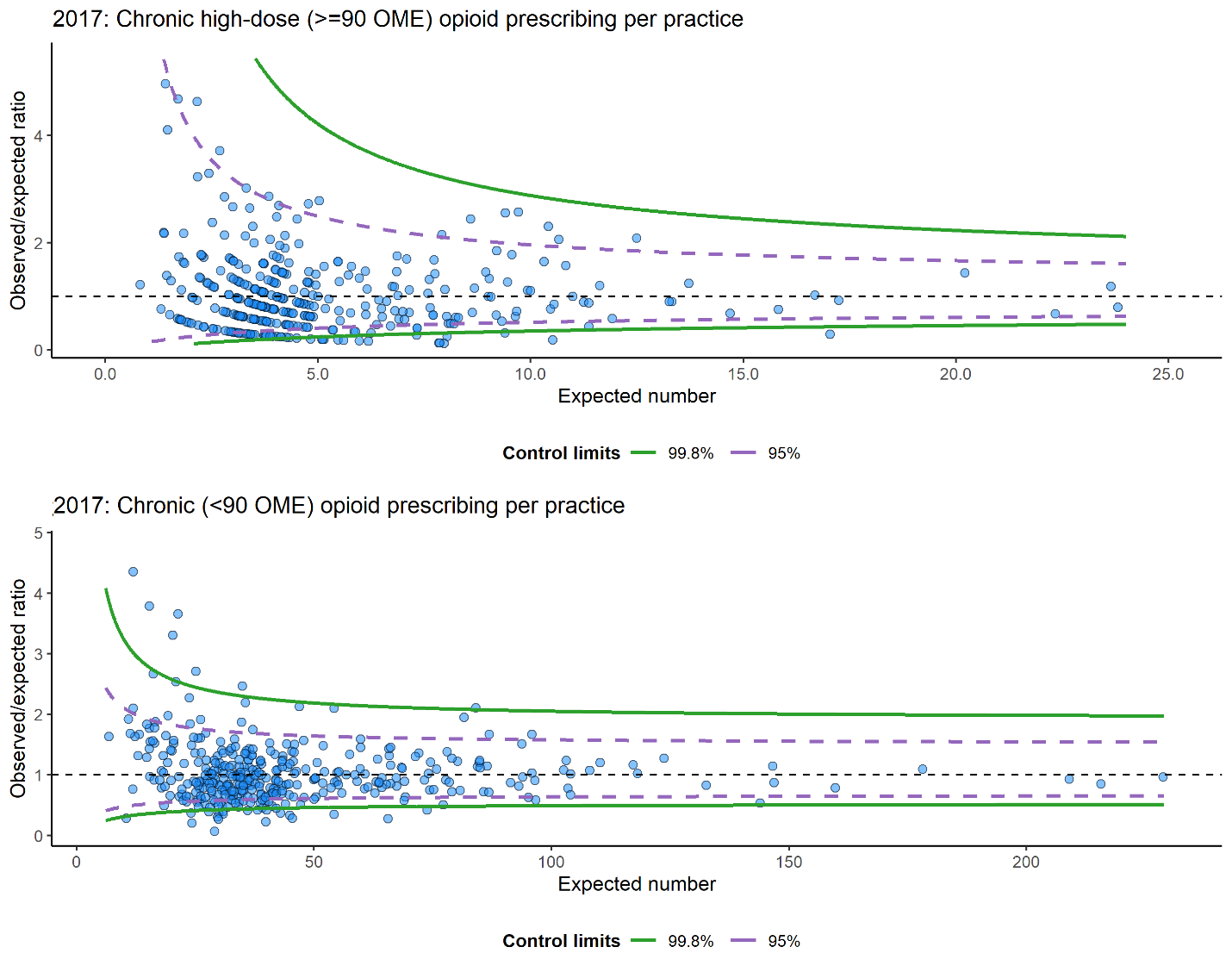


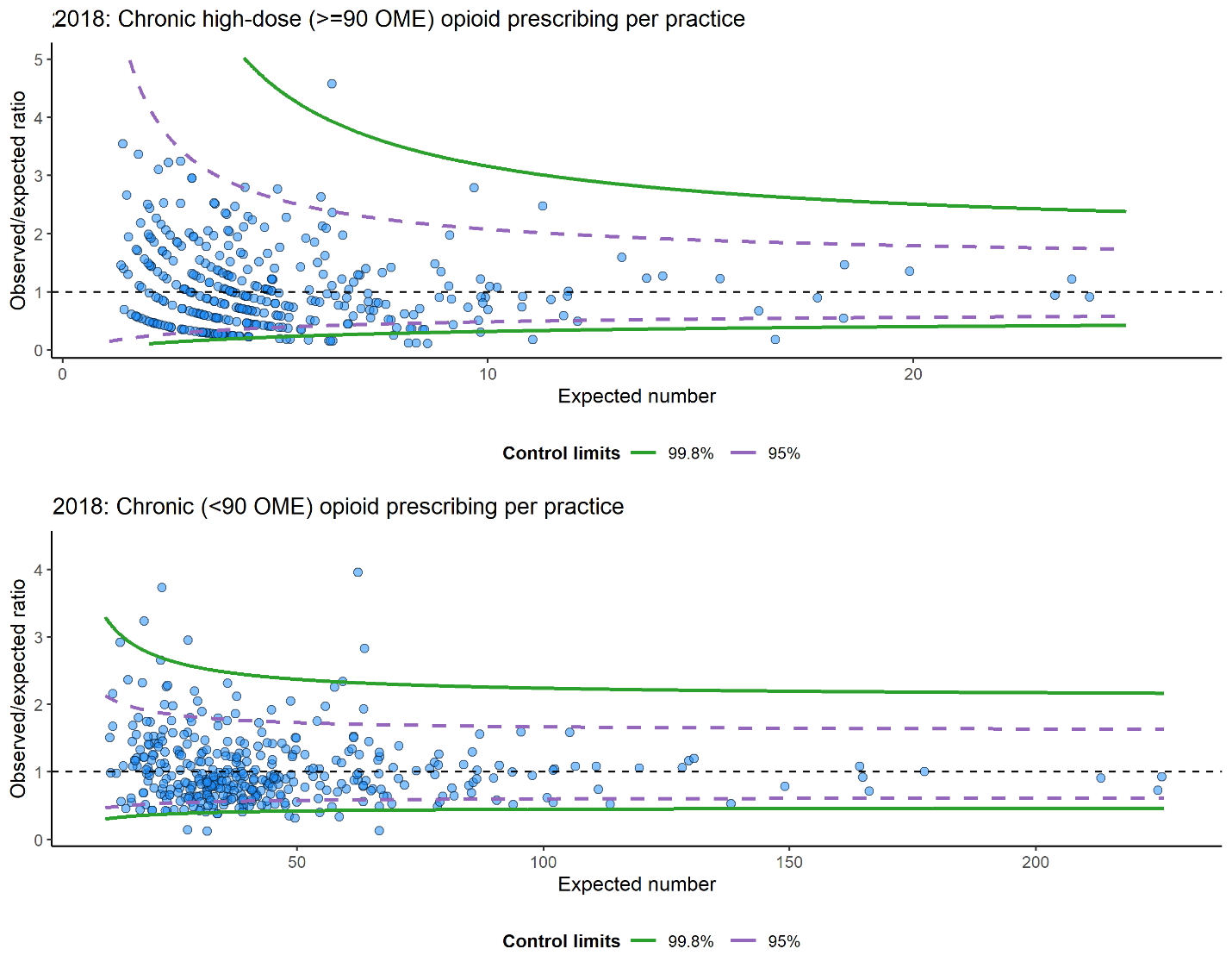

Supplement: S2 Fig — (DOCX) [file pone.0282222.s004.docx]
